# Purpose in life as a resilience factor for brain health: diffusion MRI findings from the Midlife in the U.S. study

**DOI:** 10.3389/fpsyt.2024.1355998

**Published:** 2024-03-05

**Authors:** Ajay Kumar Nair, Nagesh Adluru, Anna J. Finley, Lauren K. Gresham, Sarah E. Skinner, Andrew L. Alexander, Richard J. Davidson, Carol D. Ryff, Stacey M. Schaefer

**Affiliations:** ^1^ Institute on Aging, University of Wisconsin-Madison, Madison, WI, United States; ^2^ Waisman Center, University of Wisconsin-Madison, Madison, WI, United States; ^3^ Department of Radiology, University of Wisconsin-Madison, Madison, WI, United States; ^4^ Department of Medical Physics, University of Wisconsin-Madison, Madison, WI, United States; ^5^ Center for Healthy Minds, University of Wisconsin-Madison, Madison, WI, United States

**Keywords:** purpose in life, aging, diffusion weighted imaging, microstructure, white matter, hippocampus, MIDUS, resilience

## Abstract

**Introduction:**

A greater sense of purpose in life is associated with several health benefits relevant for active aging, but the mechanisms remain unclear. We evaluated if purpose in life was associated with indices of brain health.

**Methods:**

We examined data from the Midlife in the United States (MIDUS) Neuroscience Project. Diffusion weighted magnetic resonance imaging data (*n*=138; mean age 65.2 years, age range 48-95; 80 females; 37 black, indigenous, and people of color) were used to estimate microstructural indices of brain health such as axonal density, and axonal orientation. The seven-item purpose in life scale was used. Permutation analysis of linear models was used to examine associations between purpose in life scores and the diffusion metrics in white matter and in the bilateral hippocampus, adjusting for age, sex, education, and race.

**Results and discussion:**

Greater sense of purpose in life was associated with brain microstructural features consistent with better brain health. Positive associations were found in both white matter and the right hippocampus, where multiple convergent associations were detected. The hippocampus is a brain structure involved in learning and memory that is vulnerable to stress but retains the capacity to grow and adapt through old age. Our findings suggest pathways through which an enhanced sense of purpose in life may contribute to better brain health and promote healthy aging. Since purpose in life is known to decline with age, interventions and policy changes that facilitate a greater sense of purpose may extend and improve the brain health of individuals and thus improve public health.

## 1 Introduction

The World Health Organization defines active aging as “the process of optimizing opportunities for health, participation and security in order to enhance quality of life as people age” (1) and focuses on functional ability for healthy aging as a combination of an individual’s intrinsic capacity and interactions with relevant environmental characteristics (2). The goal of active aging is the promotion and maintenance of positive functional outcomes and experiences such as physical, mental, and social health and wellbeing throughout the aging process (3). However, individuals start their lives with differences in genetic predispositions and continue to be exposed to a wide range of non-genetic drivers of health and disease throughout their lifespan (4). Active or healthy aging therefore involves slowing down the biological aging process, even in the presence of social and environmental challenges that may impact functional abilities (5–7).

Healthy life choices, such as being physically and cognitively active, are widely known to improve health outcomes (8–10). In the face of physical decline, aging successfully can mean selecting and prioritizing goals according to their importance for optimizing gains or compensating for losses (11, 12). One predictive variable that could link a range of healthy behaviors, foster resilience in the face of adversities, and impact physical and mental health is *purpose in life* (13). Purpose in life is defined as feeling that one’s life has meaning, and having goals, intentions, and a sense of direction for one’s life (14, 15).

Mounting evidence suggests that individuals who feel a sense of purpose in life have better health and longevity, including cognitive function (16). Purpose has been reported as a potential buffer against loneliness and is associated with reduced risk of subjective cognitive decline (17), reduced risk of mild cognitive impairment and Alzheimer’s disease (18) and delayed onset of dementia and mortality (19). Purpose in life is also associated with better outcomes for physical health and behavior, including reduced risk of cardiovascular disease (20), lower systemic inflammation among men (21), lower likelihood of suicidal ideation and attempts (22), and lower odds of all-cause mortality (23, 24).

Potential mechanisms underlying these salubrious associations with purpose in life are being explored (20, 25). While it is difficult to ascribe a causal role for purpose in life in the achievement of better cognitive outcomes, post-mortem examinations suggest that purpose in life weakens the association between Alzheimer’s disease pathology and cognitive decline (26). Examination of the associations between purpose in life and brain health measures among living individuals would enable a better understanding of the underlying mechanisms.

Age related decline in brain health is well documented, as is neuroplasticity in response to cognitive training (10). White matter volume rapidly declines from the fifties onwards (27), cortical gray matter volume peaks in childhood and gradually declines through the rest of the life, whereas subcortical gray matter volume peaks during adolescence (27). One brain region that is particularly important in the study of brain health in aging is the hippocampus, due to its role in learning and memory and life-long plasticity (28). The hippocampus is one of the few brain regions where neurogenesis continues throughout adulthood (29). This subcortical structure is known to be sensitive to stress and inflammation and is one of the earliest to show age-related changes that track cognitive decline (30). The hippocampus is vulnerable to several neurodegenerative conditions and is one of the earliest brain regions affected by Alzheimer’s disease (31–33). More encouragingly, due to its highly plastic nature, this region is also sensitive to lifestyle interventions that can buffer against age-related changes in structure and function (30). Volumetric changes in brain structures reflect late-stage, accumulated changes in cellular microstructure which manifest earlier (34). Therefore, the early changes in cellular microstructure serve as valuable targets for monitoring and intervention.

Advances in diffusion magnetic resonance imaging (MRI) have enabled fine grained insight into cellular microstructure (35). This technique uses the property that water molecules exhibit random displacements in all directions (i.e., isotropic diffusion) at room temperature. The direction of movement of water molecules in brain tissue can be tracked using diffusion MRI and quantified using diffusion tensor imaging (DTI) (35). Water molecules show different degrees of hindered and restricted diffusion based on tissue type, which may be used to derive quantitative metrics to infer features of the tissue microstructure. For example, cerebrospinal fluid is present in the ventricles with very little tissue complexity; thus, high values of mean diffusivity (MD, diffusivity in all directions) would be expected. However, in a white matter bundle consisting of neuronal fibers, low radial diffusivity (diffusivity perpendicular to the fibers) is expected as water cannot freely diffuse through the myelin sheaths around the axons of neurons. Additionally, in white matter bundles, high values of fractional anisotropy (FA, diffusivity preferentially directed along fibers) are expected, such that water is diffusing more freely parallel to the axons but not in other directions. By examining the deviation of diffusion from a normal displacement distribution, complementary metrics can be derived using diffusion kurtosis imaging (DKI) (36), allowing estimation of metrics such as mean kurtosis and radial kurtosis. These kurtosis metrics are often negatively correlated with the corresponding diffusivity metrics; for example, mean kurtosis in white matter increases with decreases in mean diffusivity (37). Multiple studies have examined the validity of using diffusion MRI as a non-invasive way of tracking brain microstructural changes (38). For example, histological assessment in rabbits done along with DTI and histological assessment in human post-mortem brains done in tandem with DKI suggest that these statistical models are sensitive to tissue microstructural differences (39, 40) but they are not very specific to cellular microstructure (38).

Several advanced biophysical models, such as neurite orientation dispersion and density imaging (NODDI) and white matter tract integrity (WMTI), have been developed to better describe cellular microstructure (41, 42). These approaches model the underlying tissue microstructure to provide biologically relevant and interpretable metrics (43). For example, the NODDI model provides the neurite density index which estimates the density of axons and dendrites (collectively called neurites) and has been shown to track the integrity of axons (34). The WMTI model provides a similar metric termed axonal water fraction that captures the density of axons (44). Validation studies have been carried out using electron microscopy and histology for these biophysical models (45, 46).

Taken together, diffusion MRI metrics have been shown to be sensitive to brain microstructural changes across the lifespan, from early brain development (47) to aging and neurodegenerative conditions (37, 48). For example, diffusion MRI metrics have been shown to be sensitive to glial activation, edema, axonal swelling, and changes in axonal myelination (49). Using the complementary information provided by both the statistical models (DTI and DKI) and the biophysical models (WMTI and NODDI), we aimed to comprehensively characterize brain microstructural features associated with purpose in life, identify the most sensitive measures, and identify brain regions with converging findings that might most robustly reflect associations with purpose in life. We tested the hypothesis that higher self-reported feelings of purpose in life would be associated with whole brain white matter and hippocampal microstructural metrics consistent with better brain health.

## 2 Materials and methods

### 2.1 Study overview

This study is based on analysis of data from the third wave of the Midlife in the United States (MIDUS) study. The MIDUS study is a longitudinal national study in the United States that began in 1994 initially recruiting adults aged between 25-74 years of age and has been following them up every ten years. MIDUS seeks to understand the sociodemographic, psychosocial, and neurobiological determinants of health and illness among aging adults with the goal of advancing knowledge of resilience in the face of challenge and adversity. A unique feature of MIDUS is the focus on studying the middle segment of life and tracking individuals as they move on into old age. MIDUS data are comprehensive, with over 25000 assessed variables, and are publicly available for use by scientists around the world (https://midus.wisc.edu/).

During the third follow-up of the main MIDUS sample (MIDUS3; 2017-2022), multi-shell diffusion imaging data were acquired for the first time by the MIDUS Neuroscience Project. The present work analyzes data from those participants who underwent multi-shell diffusion weighted (DWI) MRI. All MIDUS projects were granted approval from relevant institutional review boards. All participants were briefed about study procedures and screened to ensure MRI compatibility, and all provided informed consent prior to data collection.

### 2.2 Study participants and characteristics

Participants (*n* = 138) were between the ages of 48-95 (mean 65.2, median 64, SD = 9.35) years. There were 80 females and 37 black, indigenous and people of color (BIPOC) in the sample. There were 59 participants with a college degree, 44 with some college education, and 35 with high school or less education.

### 2.3 Purpose in life assessment

Purpose in life was assessed using a seven-item questionnaire adapted from Ryff’s Psychological Well-being scale (14). This scale has seven-items such as “I live life one day at a time and don’t really think about the future”, “I enjoy making plans for the future and working to make them a reality”. Participants rated their responses to each item using a seven-point Likert scale ranging from strongly disagree to strongly agree. Negatively worded items are reverse scored so that when the sum of the items is used as a score, higher values indicate greater levels of purpose. The scale has been validated in multiple national samples (15, 50). Participants reported levels of purpose in life between 22-49 with a median score of 40.

### 2.4 Diffusion MRI data acquisition

A multi-shell spin-echo, echo-planar imaging sequence was used to collect DWI data using a Nova 32 channel head coil and a 3 Tesla GE 750 scanner. Three shells of different encoding strengths (b-values of 500, 800 and 2000 s/mm^2^) were acquired with 9, 18 and 36 directions, respectively. There were six reference scans without any diffusion encoding (b=0 s/mm^2^). Other parameters included: repetition time (TR) = 7000 ms; echo time (TE) = 91 ms; field of view (FOV) = 256 mm; 75 slices and 2x2x2 mm^3^ voxel resolution.

### 2.5 Pre-processing and estimation of brain microstructure metrics

The DESIGNER pipeline was used to pre-process the data (51). The diffusion kurtosis tensor was estimated, along with the diffusion tensor, at each voxel using weighted least-squares optimization (52). Image maps with voxel wise estimates of the DKI metrics and DTI metrics were then generated. From the estimates of the kurtosis tensor, the following WMTI model (42) parameters were computed: axonal water fraction, intra-axonal diffusivity and extra-axonal diffusivities along the axon and perpendicular to the axon. Spatially adaptive smoothing was applied to the DKI and WMTI measures following the approach used by the tissue-specific smoothing-compensated method (T-SPOON (53)) to address the spatial variation in the model estimation quality. Specifically, a brain mask was created using a threshold based on mean kurtosis (MK < 0.3). Then each DKI and WMTI metric was smoothed concurrently with the brain mask and divided by the smoothed mask to arrive at unbiased smoothed estimates. The DWI data were also used to fit the multi-tissue NODDI model (54) to derive voxel wise estimates of corresponding metrics namely neurite density index, orientation dispersion index and cerebrospinal fluid fraction. A study specific population template was created (55) using the individual subject FA and MD maps to estimate the template, and subsequently the full set of parameter maps were warped to this template space. Each set of model-specific parameter maps were grouped and merged for statistical analyses.

### 2.6 Statistical analyses and visualization

Permutation analyses of linear models (PALM) (56) were carried out to examine the association between purpose in life and microstructure metrics while adjusting for sociodemographic covariates (age, sex, race, and education). The analyses were restricted to two regions of interest using the following approach: The FA skeleton mask provided with FSL software (57) was moved to the population template space and used as a white matter mask. The bilateral hippocampal regions from the Harvard-Oxford subcortical atlas (58) were binarized and moved to the population template space to form the hippocampal mask as a region of interest due to its vulnerability to stress and neurodegeneration. Analyses were run using analytical tail acceleration and 500 permutations. Joint inference was carried out separately for DTI, DKI, WMTI and NODDI metrics using non-parametric combination and inference about each metric was simultaneously obtained. Our goal with these analyses was to examine brain health with diffusion metrics that provide both complementary and overlapping information. Therefore, we did not correct for multiple comparisons across all the diffusion metrics. Threshold-free cluster enhancement (TFCE) and family-wise error (FWE) correction across all voxels of interest were used to control for false positives for each of the diffusion metrics individually.

Voxels showing significant relationships were defined with a threshold of *p* < 0.05, corrected for multiple comparisons and corresponding statistical brain maps were generated. To visualize significant relationships of the microstructure metrics with the variables of interest, mean values across significant voxels were extracted for each participant as summarized versions of the outcome variables. Corresponding linear models were run and partial residuals were plotted for significant relationships. As the models utilize data extracted from voxels that were found significant after statistical testing, model coefficients presented in the result section are purely for descriptive purposes. Influential outliers were defined as those exceeding a threshold of 5% of the F-distribution of Cook’s distance and were removed. Two models had one outlier each, and the results were consistent with and without outlier removal. Visualizations were carried out using R statistical software (v4.3.0).

## 3 Results

### 3.1 Associations between purpose in life and whole brain white matter microstructure

**Figure 1:**
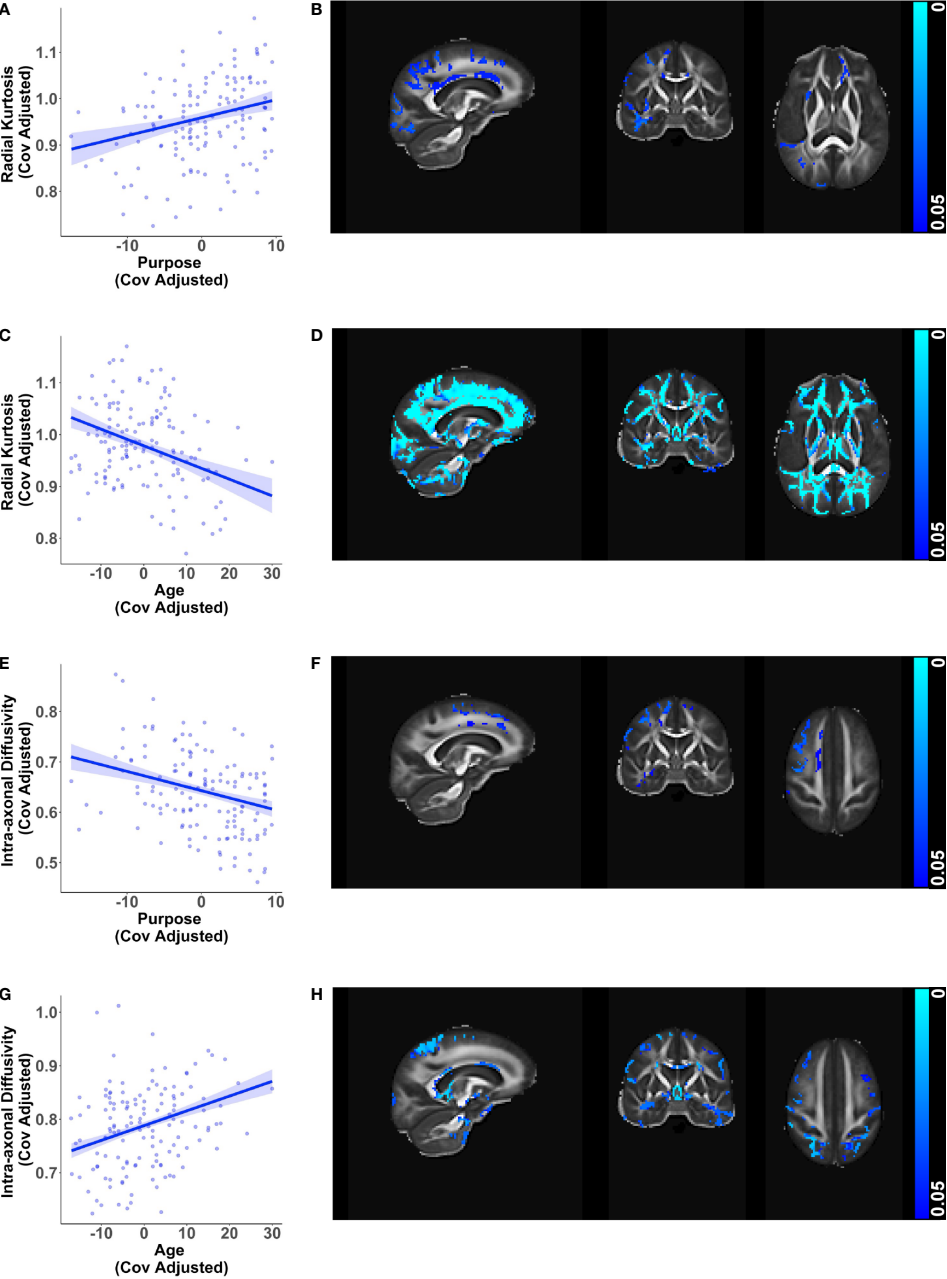
Relationships of white matter microstructure metrics with purpose in life and age. Scatter plots visualizing voxel wise relationships in whole brain white matter mask between **(A)** radial kurtosis and purpose in life, and **(E)** intra-axonal diffusivity and purpose in life. Each data point represents the mean of all significant voxels for one individual, adjusted for age, sex, education, and race. As an aid to understanding the buffering effect of purpose, corresponding scatter plots between **(C)** radial kurtosis and age, and **(G)** intra-axonal diffusivity and age are visualized, adjusted for purpose in life, sex, education, and race. For each of the above metrics, representative brain slices **(B, D, F, H)** of the population template show voxels with significant relationships (at *p* < 0.05, family wise error corrected) with the color bars indicating *p*-values. Brain images are shown in radiological convention (left hemisphere is shown on the right side in coronal and axial views).

Greater purpose was associated with higher radial kurtosis (β = 0.00388, *p* < 0.001) and lower intra-axonal diffusivity (β = -0.00383, *p* < 0.001) in the whole brain white matter mask while adjusting for age, sex, education, and race (**Figures 1A, B, E, F**). The relationships with radial kurtosis were widespread across white matter tracts, such as the anterior thalamic radiation, corticospinal tract, cingulum, forceps minor, inferior fronto-occipital fasciculus, uncinate fasciculus, superior longitudinal fasciculus, especially but not exclusively in the right hemisphere. The relationships with intra-axonal diffusivity were also present along these tracts but were more circumscribed. There was a subset of overlapping voxels, such as in the anterior thalamic radiation, that were significant for both these metrics, but the associations were predominantly in different voxels. None of the other microstructure metrics showed significant associations with purpose in life. These findings suggest a buffering effect of purpose in life against corresponding age-related changes in radial kurtosis and intra-axonal diffusivity (**Figures 1C, D, G, H**) visualized after adjusting for purpose in life, sex, education, and race.

### 3.2 Associations between purpose in life and hippocampal microstructure

**Figure 2:**
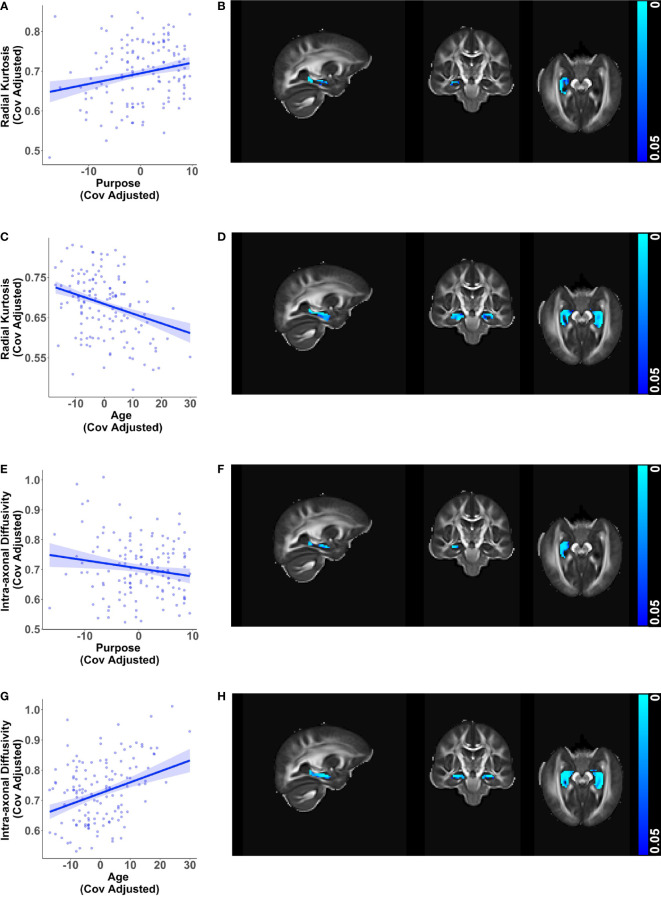
Relationships of hippocampal microstructure metrics with purpose in life and age. Scatter plots visualizing voxel wise relationships in bilateral hippocampal mask between **(A)** radial kurtosis and purpose in life, and **(E)** intra-axonal diffusivity and purpose in life race after removing influential outliers, if any. Each data point represents the mean of all significant voxels for one individual, adjusted for age, sex, education, and race. There was one influential outlier for the model with intra-axonal diffusivity. Results were consistent with and without the outlier, but the relationship became weaker after outlier removal. As an aid to understanding the buffering effect of purpose, corresponding scatter plots between **(C)** radial kurtosis and age, and **(G)** intra-axonal diffusivity and age are visualized, adjusted for purpose in life, sex, education, and race. For each of the above metrics, representative brain slices **(B, D, F, H)** of the population template show voxels with significant relationships (at *p* < 0.05, family wise error corrected) with the color bars indicating *p*-values. Significant relationships with purpose in life were localized to the right hippocampus whereas relationships with age were found in both hemispheres. Brain images are shown in radiological convention (left hemisphere is shown on the right side in coronal and axial views).

Consistent with findings in the whole brain white matter mask, greater purpose was associated with higher radial kurtosis (β = 0.00267, *p* < 0.001) and lower intra-axonal diffusivity (β = -0.00271, *p* = 0.019) in the right hippocampus while adjusting for age, sex, education, and race (**Figures 2A, B, E, F**). The directionality of these relationships with purpose in life opposed the directionality of corresponding relationships of age with radial kurtosis and intra-axonal diffusivity in the bilateral hippocampus (**Figures 2C, D, G, H**), visualized after adjusting for purpose in life, sex, education, and race. Greater purpose was also associated with higher mean kurtosis (β = 0.00179, *p* < 0.001) whereas older age was associated with lower mean kurtosis (**Supplementary Figure 1A-D**).

**Figure 3:**
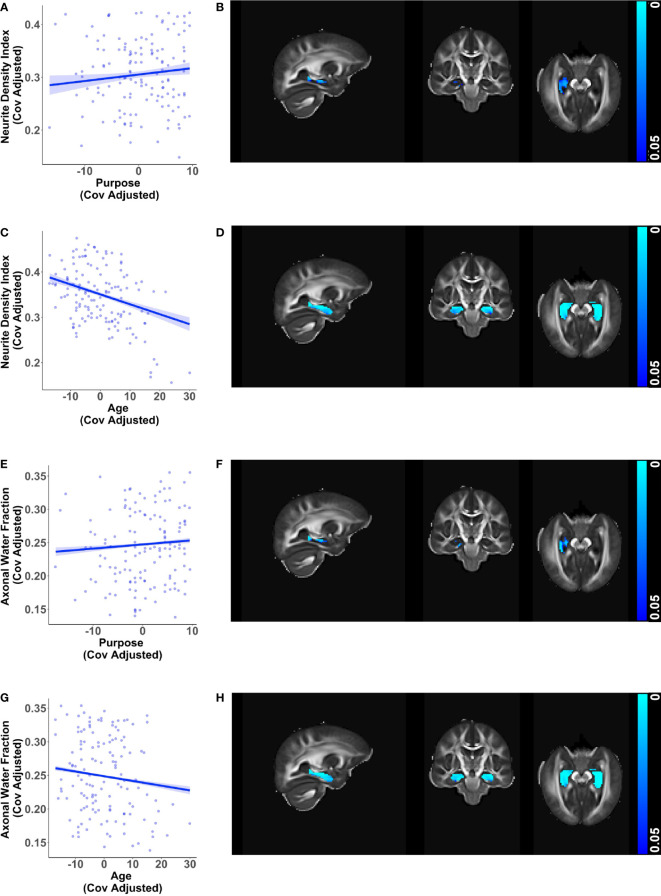
Additional relationships of hippocampal microstructure metrics with purpose in life and age. Scatter plots visualizing voxel wise relationships in bilateral hippocampal mask between **(A)** neurite density index and purpose in life, and **(E)** axonal water fraction and purpose in life after removing influential outliers, if any. Each data point represents the mean of all significant voxels for one individual, adjusted for age, sex, education, and race. There was one influential outlier for the model with neurite density index. Results were consistent with and without the outlier, but the relationship became weaker after outlier removal. As an aid to understanding the buffering effect of purpose, corresponding scatter plots between **(C)** neurite density index and age, and **(G)** axonal water fraction and age are visualized, adjusted for purpose in life, sex, education, and race. For each of the above metrics, representative brain slices **(B, D, F, H)** of the population template show voxels with significant relationships (at *p* < 0.05, family wise error corrected) with the color bars indicating *p*-values. Consistent with the other findings, significant relationships with purpose in life were localized to the right hippocampus whereas relationships with age were found in both hemispheres. Brain images are shown in radiological convention (left hemisphere is shown on the right side in coronal and axial views).

Additionally, diffusion metrics that are sensitive to changes in axonal health showed converging relationships with purpose in life and age in the hippocampus. Greater purpose was associated with higher neurite density index (β = 0.00122, *p* = 0.021) and higher axonal water fraction (β = 0.00062, *p* < 0.001) in the right hippocampus (**Figures 3A, B, E, F**), adjusting for age, sex, education, and race. In contrast, higher age was associated with lower neurite density and lower axonal water fraction in the bilateral hippocampus (**Figures 3C, D, G, H**), visualized after adjusting for purpose in life, sex, education, and race.

## 4 Discussion

The present study tested the hypothesis that higher levels of self-reported feelings of purpose in life would be associated with better brain health as indicated by whole brain white matter and hippocampal microstructural metrics. We found preliminary support for the hypothesis in whole brain white matter and more broadly in the hippocampus. Greater purpose in life was associated with higher radial kurtosis and lower intra-axonal diffusivity in both white matter and the right hippocampus. Additionally, greater purpose was associated with higher mean kurtosis, neurite density and axonal water fraction in the right hippocampus. These findings were adjusted for age, sex, education, and race, suggesting that purpose in life explained some of the variance in brain health independent of these variables that are known to be associated with brain health among aging individuals. Age is a powerful predictor for adverse microstructural differences and in our work, age showed expected relationships with the various diffusion metrics, whereas purpose in life showed opposing relationships consistent with our hypothesis of its role as a resilience factor for brain health.

Radial and mean kurtosis values are known to be negatively correlated with age in both white and gray matter in midlife (59). Among older adults, radial and mean kurtosis values were found to be lower in mild cognitive impairment and Alzheimer’s disease, as compared to controls, in both white matter regions and in the hippocampus (37, 60). We replicated these findings of negative relationships of radial and mean kurtosis with age, confirming that these metrics are sensitive to age associated decline in brain health. Thus, the higher values of mean and radial kurtosis found in association with greater purpose are suggestive of better brain health.

Intra-axonal diffusivity is a marker of axonal injury, and is decreased with axonal beading (focal enlargements separated by constrictions) in response to ischemic stroke (44, 61). However, this metric was not found to be effective in differentiating between controls and patients with Alzheimer’s disease, suggesting that disease progression along the Alzheimer’s disease continuum is less dependent on intra-axonal environment (44). In our sample, age was positively associated with higher intra-axonal diffusivity in both white matter and the hippocampus, indicating age-related changes in the intra-axonal environment. Because purpose was negatively associated with this metric after adjusting for age and other covariates, greater purpose may buffer against age-related changes in the intra-axonal environment.

Although the association between purpose in life with radial kurtosis and intra-axonal diffusivity were widespread in whole brain white matter, there were few regions with a strong overlap. This is not unexpected, as radial kurtosis is sensitive to microstructural differences but is not based on a biophysical model, unlike the intra-axonal diffusivity which, is more specific and biologically interpretable. One of the reasons for employing multiple diffusion metrics in our study was to identify measures that are more sensitive to our question of interest and to examine overlap and complementarity. Further research is needed to replicate and to investigate the effects of purpose on these microstructural indices.

The association of purpose in life with several diffusion metrics showed consistent and converging results suggestive of better microstructural integrity in the right hippocampus. Axonal water fraction is a marker of axonal integrity from the WMTI model (45). Lower axonal water fraction may suggest reduction in axons in response to cortical atrophy (44, 62). Neurite density index is a closely related axonal marker from NODDI that shows similarity with myelin maps (63). In our sample, even after adjusting for covariates, these metrics exhibited a positive association with purpose in life. This suggests that participants with greater levels of purpose may have better-preserved myelinated axons.

The consistent laterality in findings of associations of purpose in life with the right hippocampus is intriguing. Hemispheric differences in neuronal morphology and density between the hippocampi may underlie differences in memory function and subserve vulnerability to disease (64). Interestingly, in a spatial navigation paradigm, activation of the right hippocampus was predictive of other-centric spatial representation, whereas activation of the left hippocampus predicted the use of a self-centric representation (65). Additional findings linking the right hippocampus with spatial processing whereas the left with verbal and autobiographical processing (66, 67) suggest the left and right hippocampi may play different functional roles or at least be involved to different degrees in some cognitive functions. Our finding of higher levels of purpose in life being associated with better microstructural metrics in the right hippocampus need to be investigated further to better understand this lateralized finding and its implication for cognitive functioning.

While diffusion tensor and kurtosis metrics are quite sensitive to changes in microstructure, they are difficult to interpret in terms of changes in the underlying biology (35, 45). Variations of the standard model of microstructure such as NODDI and WMTI partial out each voxel into multiple compartments and provide more interpretable information (68). For example, although DTI and NODDI metrics both showed age related declines in the hippocampus, the NODDI model measures showed stronger relationships and explained more of the age-related variance (69). Each of these approaches make assumptions that need to be considered. For example, NODDI is known to overestimate the neurite density index, and cerebrospinal fluid fraction in white matter (70). Since kurtosis metrics and related estimates are susceptible to noise artifacts and confounds, we used adaptive smoothing to overcome the black hole related shrinkage bias that occurs when smoothing DKI and WMTI metrics. Newer improved models are being developed to try and address limitations (71, 72). The converging findings from these different DWI models present intriguing evidence of their applicability in the hippocampus and similar regions that needs to be tested further.

What might be the mechanisms underlying the positive relationship of purpose in life with the various indices of microstructural health? A review of the literature suggested that greater feelings of purpose might mitigate health risks by enhancement of other psychological and social resources that buffer against stress, by indirect effects by modification of health-related behaviors, and potentially by direct influence on biological pathways (20). For example, in the Rush Memory and Aging Project, lower levels of purpose in life have been associated with greater loneliness, more anxiety-related harm avoidance, older age and more depressive symptoms, whereas higher levels of purpose in life are associated with greater perceived social support, more social activities, more years of education, higher income, more intact cognition in late-life, and more middle-age cognitive activities (25). Collectively, these associations suggest modifiable targets for intervention to enhance feelings of purpose in life, which may in turn enhance brain health.

Purpose in life has been defined as “a central, self-organizing life aim that organizes and stimulates goals, manages behaviors, and provides a sense of meaning” (13). Other theorists have defined purpose as “a stable and generalized intention to accomplish something that is at once meaningful to the self and of consequence to the world beyond the self” (73). In the present work, we define purpose as a feeling that one’s life has meaning, and having goals, intentions, and a sense of direction for one’s life (14, 15). This sense of leading a meaningful and worthwhile life is associated with better lifestyle choices such as exercise and diet, sleep quality, better mental health and physical fitness, stronger personal and social relationships, prosocial activities such as volunteering and even greater financial outcomes (74). Several of these behaviors are related to better brain health (75, 76), suggesting that feelings of purpose in life might encourage healthier lifestyle choices that buffer against stress and promote resilience to adversity. More directly, purposeful activity may facilitate experience dependent changes in brain circuitry related to efficient decision making, alter reactivity to emotional stressors and more broadly, enable healthier self-regulation (77). Although our analyses examining associations with a self-report measure and cross-sectional data do not permit strong claims about the mechanisms, the diffusion MRI models offer insights into the probable neurobiological pathways through which purpose may be exerting salubrious effects. Our findings point toward alterations in axonal health and myelination, although a limitation with our current findings is that the most robust effect was found in the hippocampus, where these metrics are less straightforward to interpret. Replication of our findings with larger samples is warranted. The next wave of follow-up MIDUS data collection is already underway, providing future opportunities for longitudinally tracking the relationship between purpose in life, cognition, and diffusion metrics of brain health.

In general, purpose in life develops until mid-life and subsequently declines with age (14, 78), although the benefits of preserved purpose in life in old age remain relevant (79). There is also evidence that individual trajectories in the sense of purpose stay resilient to the onset of health adversities (80). It is noteworthy that adversities such as cognitive dysfunction and dementia in late life are preceded by a long period of pre-symptomatic accumulation of pathologies (81). Given the evidence that purpose in life may delay progression to dementia and mortality by several years (19), the time gap between initial detection of brain pathologies and onset of symptoms represents a last window of opportunity for the application of effective interventions that can make a profound impact on personal and public health. Effective interventions to facilitate greater purpose in life are therefore very important throughout the life span.

Indeed, there is evidence that purpose can be developed across the life-course. The 4-H program and development of frameworks such as the pedagogy of care using processes such as exploration, engagement, reflection, articulation, and actualization may support development of purpose among youth (82, 83). Interventions to clarify values and develop purpose in life are now freely available (77) and, more broadly, interventions for psychological well-being are available at scale with refinements being considered to provide meaningful effects (84). The recent call to consider prosociality, or positive other-regarding behaviors and beliefs, as a public health priority (85) would benefit from interventions that facilitate clarification of values and foster development of purpose at the individual and societal level (86).

In summary, we found evidence that greater sense of purpose in life is associated with indices of brain microstructure suggestive of better brain health, even when accounting for age, sex, race, and education. These associations were strongest in the right hippocampus, a brain structure that is both vulnerable to stress but is highly plastic. Our findings suggest that greater sense of purpose in life may support better brain health and add to the accumulating evidence recognizing a need for interventions to augment and maintain the sense of purpose for healthy and active aging, including brain health.

## Data availability statement

The original contributions presented in the study are included in the article/**Supplementary Material**. All MIDUS data are archived and made publicly available—and are thus share-able—via the University of Michigan Inter-university Consortium of Political and Social Research (ICPSR, https://www.icpsr.umich.edu/web/ICPSR/series/203), the MIDUS Portal (https://midus.colectica.org/), or via restricted access to the MIDUS Neuroscience repository (see https://www.midus.wisc.edu/midus_neuro_data.php/ for how to obtain access). Further inquiries can be directed to the corresponding author. 

## Ethics statement

The studies involving humans were approved by University of Wisconsin-Madison. The studies were conducted in accordance with the local legislation and institutional requirements. The participants provided their written informed consent to participate in this study.

## Author contributions

AN: Investigation, Methodology, Software, Visualization, Writing – original draft, Writing – review & editing. NA: Investigation, Methodology, Software, Writing – review & editing. AF: Investigation, Writing – review & editing. LG: Data curation, Project administration, Writing – review & editing. SES: Data curation, Writing – review & editing. AA: Investigation, Methodology, Writing – review & editing. RD: Funding acquisition, Writing – review & editing. CR: Funding acquisition, Writing – review & editing. SMS: Conceptualization, Funding acquisition, Investigation, Supervision, Writing – review & editing.
