# Supplementary material for: Purpose in life as a resilience factor for brain health: diffusion MRI findings from the Midlife in the U.S. study
Source: Front Psychiatry. 2024 Mar 5;15:1355998. doi: 10.3389/fpsyt.2024.1355998 (PMC10948414; doi:10.3389/fpsyt.2024.1355998)
Supplement: Supplementary file 1 [file DataSheet_1.docx]

Supplementary Material

# Supplementary Figure


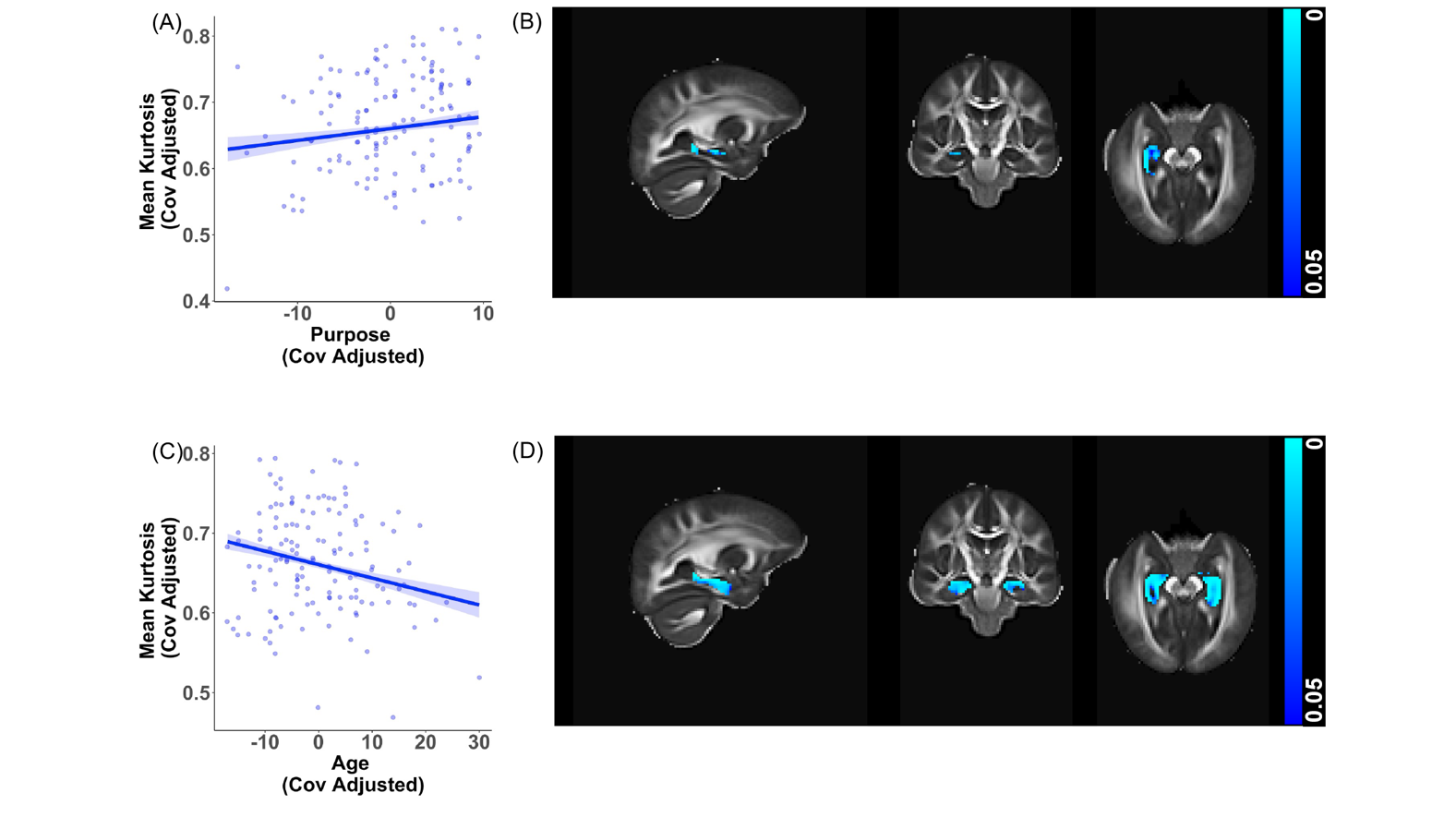


**Supplementary Figure 1: Relationships of mean kurtosis with purpose in life and age in the bilateral hippocampus.** Scatter plots visualizing the relationships of (A) mean kurtosis with purpose in life and (C) mean kurtosis with age. Each data point represents the mean of all voxels for one individual. The model for purpose in life adjusted for age, sex, education and race, and the model for age adjusted for purpose in life, sex, education, and race. Corresponding representative brain slices (B, D) of the population template show voxels with significant relationships (at *p* < 0.05, family wise error corrected) with the color bars indicating *p*-values. Consistent with the other findings, significant relationships with purpose in life were localized to the right hippocampus whereas relationships with age were found in both hemispheres. Brain images are shown in radiological convention (left hemisphere is shown on the right side in coronal and axial views).
